# Editorial: Suicide prevention in the educational context

**DOI:** 10.3389/fpsyg.2024.1497113

**Published:** 2024-10-16

**Authors:** Janaina Minelli De Oliveira, Fabia Morales-Vives, Carla D. Chugani, Maria Jimenez-Herrera, Jorge-Manuel Dueñas

**Affiliations:** ^1^Pedagogy Department, Universitat Rovira i Virgili, Tarragona, Spain; ^2^Psychology Department, Research Center for Behavior Assessment, Universitat Rovira i Virgili, Tarragona, Spain; ^3^Mantra Health, New York, NY, United States; ^4^Department of Nursing, Universitat Rovira i Virgili, Tarragona, Spain

**Keywords:** suicide prevention, learning, education, mental health, interdisciplinary research

Given the prevalence of suicidal behavior among young people, the educational context has become crucial for the development, implementation and evaluation of prevention interventions. However, there is a strong need for research that focuses on the opportunities and needs within this context, bringing together education and health sciences. For this reason, this Research Topic examines the central role of educational institutions by integrating findings from seven groundbreaking studies, highlighting challenges, successes and future directions.

Pre-college interventions are addressed in two studies. Jenniskens et al. focus on a multimodal, school-based approach for depression and suicide prevention in adolescents called STORM. The authors use an implementation mapping protocol that combines theory and co-creation with stakeholders from education, the Public health Service, mental health services, and municipalities. They encourage close collaboration with practice and consideration of long-term sustainability, among other issues. The other article, by Biber and Brandenburg, describes a community case initiative called “College Adopt-A-School”. It is an evidence-based program that uses Sources of Strength campaigns to promote protective factors to reduce suicide and opioid use. More specifically, university undergraduates are trained to implement this program alongside high school peer leaders for 2 years. The buy-in and willingness of the high school to coordinate the implementation of the program, and the ability of the university leadership team to overcome barriers, were identified as key success factors. The program implementation was deemed successful based on its adherence to the protocol, the collaboration between high school and university students, or the peer to high school student body ratio numbers, among other factors.

Two articles address incidence of suicide-related behaviors among international students. These students face a unique combination of challenges such as language barriers, cultural adjustment, financial stress, and academic pressure, and are less likely to seek help for mental health-related struggles. All these circumstances may potentially increase the risk of suicidal thoughts and behaviors. Veresova et al.'s Systematic Review article examines the prevalence rates, and risk and protective factors, for suicidal ideation, attempts, and self-harm in international students. The authors present a list of recommendations relevant for International student offices and their staff and suggest that more high-quality research featuring robust measurement is needed to adequately capture the prevalence of suicide-related outcomes in international students, particularly via longitudinal designs to establish causal relationships.

McKay and Meza's Conceptual Analysis article addresses limitations of the Western-centric strategies for suicide prevention, focusing on international students. The cultural contextualization is highlighted as a crucial component for effective prevention and intervention in these heterogenous students. The authors propose that integrating culturally contextualized prevention models, which blend evidence-based Western mental health best practices with the unique cultural perspectives of the international student population, can enrich existing efforts and unveil innovative opportunities for prevention and intervention. This approach proposes a fundamental shift in conceptualizing and approaching suicide prevention, emphasizing cultural strengths and resources over deficit-focused models.

Three articles address suicide-related themes related to medical students, a vulnerable group. Schulz et al.'s Mini Review article addresses the lack of formal training medical students and physicians receive about suicide intervention and how this lack of training may play a significant role in influencing negative attitudes, leading to a lack of care for patients. Effective intervention training to prepare primary care providers is presented by Schulz et al. as a top area where improvements can prevent suicides.

Zheng et al. original research article' findings suggest that pre-COVID-19 short sleep duration and eveningness chronotype are significantly associated with an increased risk of incident suicide ideation (SI) during the COVID-19 pandemic in medical students. Their results indicate that self-reported short sleep duration is a predictor for future SI, no matter whether in common situations or under major health events. Therefore, prolonging sleep by increasing sleep opportunities might be helpful.

Lastly, but not least, Wu et al.'s Original Research article regarding the risk of suicidal behaviors medical students suggest that providing mental health training and building healthy student-supervisor relationships can be supportive. They suggest establishing professional courses for both students and supervisors to regulate the rights and obligations in their relationship. Attention to medical students from families with low socioeconomic status and encouragement and guidance to these students are needed to help them to communicate with supervisors more proactively.

